# Pyrosequencing analysis of a bacterial community associated with lava-formed soil from the Gotjawal forest in Jeju, Korea

**DOI:** 10.1002/mbo3.238

**Published:** 2015-01-21

**Authors:** Jong-Shik Kim, Keun Chul Lee, Dae-Shin Kim, Suk-Hyung Ko, Man-Young Jung, Sung-Keun Rhee, Jung-Sook Lee

**Affiliations:** 1 Gyeongbuk Institute for Marine BioindustryUljin, 767-813, Korea; 2 Korea Research Institute of Bioscience and BiotechnologyDaejeon, 305-806, Korea; 3 Research Institute for HallasanJeju Special Self-Governing Province, 690-816, Korea; 4 Department of Microbiology, Chungbuk National UniversityCheongju, 361-763, Korea

**Keywords:** 16S rRNA gene, bacterial diversity, Gotjawal forest soil, pyrosequencing

## Abstract

In this study, we analyzed the bacterial diversity in soils collected from Gyorae Gotjawal forest, where globally unique topography, geology, and ecological features support a forest grown on basalt flows from 110,000 to 120,000 years ago and 40,000 to 50,000 years ago. The soils at the site are fertile, with rocky areas, and are home to endangered species of plants and animals. Rainwater penetrates to the groundwater aquifer, which is composed of 34% organic matter containing rare types of soil and no soil profile. We determined the bacterial community composition using 116,475 reads from a 454-pyrosequencing analysis. This dataset included 12,621 operational taxonomic units at 3% dissimilarity, distributed among the following groups: *Proteobacteria* (56.2%) with 45.7% of *α-Proteobacteria*, *Actinobacteria* (25%), *Acidobacteria* (10.9%), *Chloroflexi* (2.4%), and *Bacteroidetes* (0.9%). In addition, 16S rRNA gene sequences were amplified using polymerase chain reaction and domain-specific primers to construct a clone library based on 142 bacterial clones. These clones were affiliated with the following groups: *Proteobacteria* (56%) with 51% of *α-Proteobacteria*, *Acidobacteria* (7.8%), *Actinobacteria* (17.6%), *Chloroflexi* (2.1%), *Bacilli* (1.4%), *Cyanobacteria* (2.8%), and *Planctomycetes* (1.4%). Within the phylum *Proteobacteria*, 56 of 80 clones were tentatively identified as 12 unclassified genera. Several new genera and a new family were discovered within the *Actinobacteria* clones. Results from 454-pyrosequencing revealed that 57% and 34% of the sequences belonged to undescribed genera and families, respectively. The characteristics of Gotjawal soil, which are determined by lava morphology, vegetation, and groundwater penetration, might be reflected in the bacterial community composition.

## Introduction

**Figure 1:**
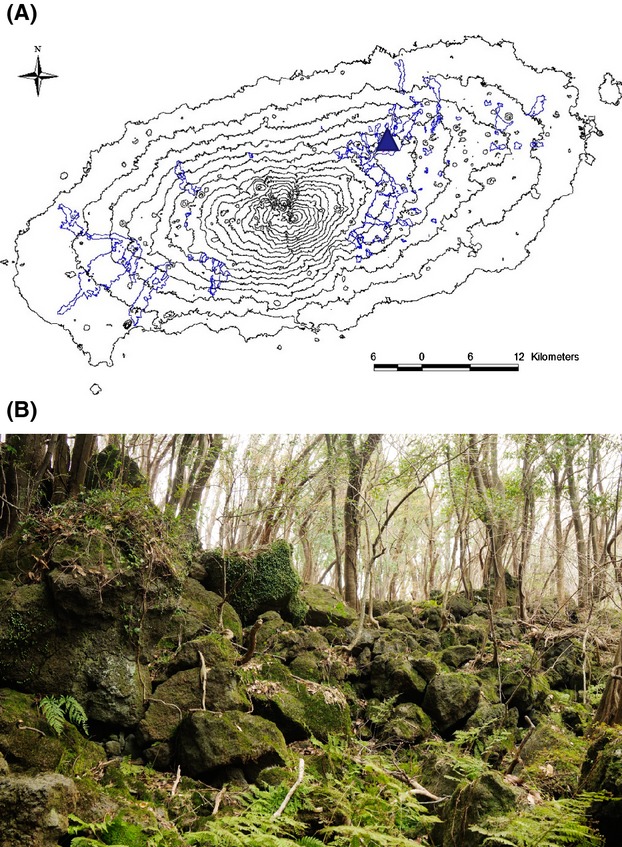
(A) A map of Jeju Island showing the Gotjawal distribution (blue line) and the sampling site (blue triangle symbol). (B) Photographs showing the Gyorae Gotjawal forest in Jeju (N 33° 26′ 24.9″ and E 126° 39′ 44.5″, Korea). Pahoehoe lava and a'a lava were mixed when the lava flowed. The lava-formed forest was developed, with characteristic topography including lava domes and depressions. Vegetation in this area is mainly deciduous broadleaf trees, *Styrax japonicus*, *Zelkova serrata*, Thunb *Makino*, and *Acer palmatum* Thunb. ex Murray (Maple). Within the herb layer, *Arachniodes standishii* (T. Moore) *Ohwi* is the predominant species.

On Jeju Island in Korea, the word “Gotjawal” refers to any naturally formed forest that grows on basalt-flow rocky terrain and presents a virtually impassable mixture of trees and undergrowth. In addition, these forests function as the main source of water for Jeju's population; rainwater is purified and recharged by the porous rocks and groundwater aquifers within the forest. Gotjawal forests are characterized by lava domes, microclimates, and ecological features shaped by volcanic activity occurring 110,000–120,000 and 40,000–50,000 years ago (Park 2010). Overall, the Gotjawal forest represents a species-rich ecosystem of coexisting plant species, such as ferns and broad-leaved trees, at both the northern and southern distributional limits (Yang et al. 2006; Kim et al. 2010), harboring a total of 506 plant and 784 insect species identified to date (Jung 2010; Kim et al. 2010). The forest occurs on a highly irregular substrate of a'a lava flows (Fig.1). Jeju's lava forests may be a globally unique area and critical for understanding lava-formed forests. However, many regions of the Gotjawal forest have been deforested, used for charcoal and edible mushroom production, and grazed by horses, cows, and other herbivores. In response to these insults, secondary forests have developed on disturbed sites. Nonetheless, these forests have been gradually disappearing in recent decades, and approximately 50% of these forests have been destroyed; currently, only about 6% of the original forest area remains due to unregulated construction and urbanization (Jung 2009).

Until recently, few studies had characterized the microorganisms in Gotjawal forest soils; therefore, microbial analyses are necessary to understand the microbial communities within these soils and for elucidating the characteristics that permit the formation of these microbial communities (Kim et al. 2014a). Generally, soil microbial communities are highly diverse, and estimates of unclassified species may reach 99% of 16S rRNA gene sequence databases, since most soil bacteria are difficult to cultivate (Torsvik et al. 1990; Amann et al. 1995).

Microbial communities associated with volcanoes have been studied in the lava flow soils, rocks, and/or glass found at Kilauea volcano, Hawaii (King 2003; Dunfield and King 2004; Nanba et al. 2004; Gomez-Alvarez et al. 2007; King and Weber 2008; Nacke et al. 2011; King and King 2012); Mauna Loa, Hawaii (Crews et al. 2001); Miyake Island, Japan (Ohta et al. 2003); Llaima volcano, Chile (Hernandez et al. 2014); and Mt. Hekla, Iceland (Kelly et al. 2010). Cutler et al. (2014) suggested that plant community composition is a significant determinant for fungal communities, but is less relevant for bacterial community composition during long-term changes in soil microbial communities. Bacteria are able to colonize recent volcanic deposits, which can contain numerous unknown bacterial species (Gomez-Alvarez et al. 2007). Various aspects of the structure and function of microbial communities have been studied in recent Hawaiian volcanic deposits (Dunfield and King 2004), and these deposits in particular have been shown to harbor very distinct microbial assemblages. Three hundred-year-old lava-derived forest soils have been shown to exhibit substantial diversity (Nusslein and Tiedje 1998).

In this study, we analyzed the composition and diversity of bacteria in Gotjawal soils using 454-pyrosequencing and polymerase chain reaction (PCR) cloning-based approaches. The results of our study will provide important insights into the understanding of the soil microbial community in lava forest soils.

## Materials and Methods

### Collection of soil samples in Gotjawal

The geographic coordinates of the sample collection site were 33° 26.023′ N and 26° 39.46′ W (Gyorae Gotjawal; Fig.1A and B). In May 2009, samples of soil from behind or between the lava and trees were collected aseptically using ethanol-disinfected spatulas. Samples were placed in clean, sealable plastic bags. These samples were stored in a cooler during transfer to the laboratory and were then stored at 5°C for 1 week until further processing. After sieving (using a 2-mm sieve), subsamples were frozen at −70°C. Soil DNA extraction was conducted within 1 week of collection. The soil samples had a pH of 4.5, electrical conductivity of 3.44 dS/m, organic matter content of 34%, and NO_3_^−^ concentration of 300.48 mg/kg dry soil (Kim et al. 2014a).

### Soil DNA extraction

DNA was directly extracted from three subsamples using a FastDNA SPIN kit for soil (QBiogene Inc., Vista, CA) according to the manufacturer's protocol. The extracted DNA was purified using a FastPure DNA kit (Takara Bio Inc., Shiga, Japan) and concentrated using a Zymoclean gel DNA recovery kit (Zymo Research Corp., Orange, CA). The purified DNA from 20 subsamples was then combined and used to generate amplicons for 454-pyrosequencing and for construction of the clone library.

### 454-Pyrosequence analysis

Detailed information for the 454-pyrosequencing has been described previously (Finkel et al. 2012). Bacterial 16S rRNA gene amplicons were generated using the universal primers 27F (5′-GAG TTT GAT CMT GGC TCA G-3′) and 800R (5′-TAC CAG GGT ATC TAA TCC-3′) flanking V1–V3 hypervariable regions in the small-subunit rRNA gene. These amplicons were pyrosequenced using a Roche 454 GS-FLX pyrosequencer with Titanium reagents (Macrogen, Seoul, Korea) according to the manufacturer's instructions.

### Clone library analysis

Detail information for the cloning and transformation has been previously published (Kim et al. 2012). The primers used to amplify the 16S rRNA genes for bacteria were 27F and 1492R (Lane 1991). The amplification conditions for the PCR were 95°C for 5 min, followed by 30 cycles of 95°C for 45 sec, 55°C for 45 sec, and 72°C for 90 sec, with a final extension step for 5 min at 72°C. PCR products were purified with a QIAquick PCR purification kit (Qiagen, Valencia, CA) and ligated into a pUC118 HincII/BAP vector (Takara Bio Inc.), which was transformed into competent *Escherichia coli* DH5*α* cells (Invitrogen Corp., Carlsbad, CA) using heat shock. Plasmids from *E*. *coli* DH5*α* transformants were isolated using the PureLink Quick Plasmid Miniprep kit (Invitrogen Corp.). The 16S rRNA genes from the bacterial clones were sequenced using an Applied BioSystems model 3730xl automated DNA sequencing system (Foster City, CA).

### Data analysis

#### Pyrosequencing

**Figure 2:**
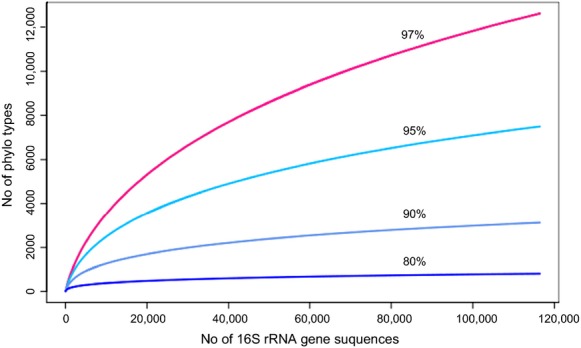
Rarefaction curves of 454-pyrosequences indicating the observed number of OTUs in the Gyorae Gotjawal forest soil.

For the determination of operational taxonomic units (OTUs), we defined species, genus, family, and phylum levels at 3%, 5%, 10%, and 20% dissimilarity, respectively, following the procedures used by Schloss and Handelsman (2005). For taxonomy-based analysis, the SILVA database (Pruesse et al. 2007) was used with the unique sequences at an 80% confidence threshold cutoff. Rarefaction curves were analyzed using the R VEGAN package (Oksanen et al. 2011; Fig.2), and richness estimates were analyzed using MOTHUR (Schloss et al. 2009). Short reads (26,382 reads) less than 289 bp and reads with ambiguous nucleotides (17,730 reads) were removed from analysis. Long reads more than 724 bp were trimmed using CD-Hit-OTU (Li et al. 2012). Chimeric sequences (1091 reads) were removed using MOTHUR. Reads from all datasets were quality filtered using a Q20 quality cutoff.

#### Clone library analysis

Putative chimeric sequences were identified using Bellerophon (Huber et al. 2004). The 16S rRNA gene sequences were aligned using the Nearest Alignment Space Termination (NAST) aligner (DeSantis et al. 2006a), and the aligned sequences were compared to the Lane mask using the Greengenes website (DeSantis et al. 2006b). The Sequence Match feature of RDPII (Cole et al. 2009) was used to find GenBank sequences representing the most closely related type strain for each clone, which were then included as references in the phylogeny. Using the Greengenes Automatic Taxonomic Classification algorithm (DeSantis et al. 2006a) and GenBank, a set of related sequences was interpreted as a novel genus (or species) if they were classified as the same genus (or species).

Phylogenetic trees were constructed using neighbor-joining with MEGA version 5.0 for Windows (Tamura et al. 2011). Evolutionary distances were calculated using the Kimura 2-parameter method (Kimura 1980). Bootstrap analyses of the neighbor-joining data were conducted based on 1000 samples to assess the support for inferred phylogenetic relationships. DOTUR (Schloss and Handelsman 2005) was used to calculate taxon richness and diversity estimates. A distance matrix was obtained using the Calculate Distance Matrix algorithm from the Greengenes website (DeSantis et al. 2006a,b).

### Nucleotide sequence accession numbers

All pyrosequencing reads were deposited in the DDBJ Sequence Read Archive (SAR) under the study accession number DRP002233. Bacterial clone sequences were deposited in the DDBJ under the following accession numbers: AB821051–AB821192.

## Results and Discussion

### Analysis of bacterial sequences

**Figure 3:**
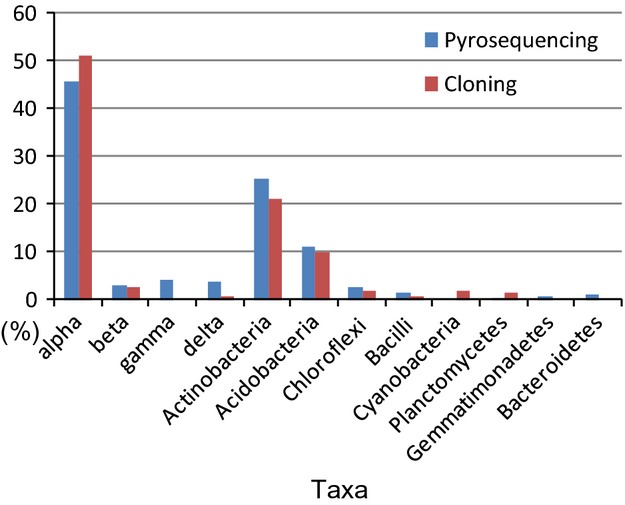
Phylogenetic distribution of operational taxonomic units (OTUs) and 454-pyrosequencing/clones observed in Gotjawal soil.

In this study, we examined a total of 116,475 reads and 142 clones representing 10 and 7 phyla, respectively, from Gotjawal forest soil. Figure3 and Table1 summarize the phylogenetic distribution of the 454-pyrosequences and clone sequences of the 16S rRNA gene. The class *α-Proteobacteria*, and phyla *Actinobacteria* and *Acidobacteria* dominated the bacterial community in the soil, representing 45.6%, 25.2%, and 10.9% of the 454-pyrosequences, respectively. The same phyla or classes dominated the clone library, representing 51.2%, 21%, and 10% of sequences, respectively.

**Table 1:** Relative abundances of the phylogenetic groups in Gotjawal soil

Phylum/Class		Order		Family		Genus	
*Proteobacteria*	56.4						
*Alpha*	45.7	*Rhizobiales*	30.2	*Hyphomicrobiaceae*	3.2	*Pedomicrobium*	1.7
						*Rhodomicrobium*	0.3
						*Blastochloris*	0.5
						*Rhodoplanes*	0.3
						*Prosthecomicrobium*	0.2
						*Rhodomicrobium*	0.3
						*Hyphomicrobium*	0.2
				*Xanthobacteraceae*	16.3	*Pseudolabrys*	0.9
						*Labrys*	0.4
				*Rhodobiaceae*	1.0	*Rhodobium*	0.6
				*Bradyrhizobiaceae*	4.8	*Bradyrhizobium*	4.2
						*Afipia*	0.1
				*Rhizobiaceae*	0.6	*Rhizobium*	0.6
		*Rhodocyclales*	0.2	*Rhodocyclaceae*	0.2	*Azospira*	0.2
		*Sphingomonadales*	0.1	*Sphingomonadaceae*	0.1	*Sphingomonas*	0.0
		*Caulobacterales*	0.6	*Caulobacteraceae*	0.6	*Phenylobacterium*	0.1
*Beta*	2.9	*Burkholderiales*	1.8	*Burkholderiaceae*	1.2	*Burkholderia*	1.2
				*Comamonadaceae*	0.4	*Variovorax*	0.2
*Gamma*	4.1	*Pseudomonadales*	0.1	*Pseudomonadaceae*	0.1	*Pseudomonas*	0.1
		*Xanthomonadales*	3.4	*Xanthomonadaceae*	0.6	*Dyella*	0.2
		*Legionellales*	0.4	*Coxiellaceae*	0.4	*Aquicella*	0.3
*Delta*	3.5	*Myxococcales*	1.8	*Kofleriaceae*		*Haliangium*	0.7
				*Polyangiaceae*	0.4	*Sorangium*	0.3
*Acidobacteria*	10.9	*Acidobacteriales*	4.0	*Acidobacteriaceae*	4.0	*Candidatus Solibacter*	2.6
						*Edaphobacter*	0.6
						*Acidobacterium*	0.4
				*Unclassified*		*Bryobacter*	0.8
						*Candidatus Koribacter*	0.3
*Actinobacteria*	25.2	*Actinomycetales*	17.5	*Acidothermaceae*	11.5	*Acidothermus*	11.5
				*Mycobacteriaceae*	2.0	*Mycobacterium*	2.0
				*Streptomycetaceae*	1.0	*Streptomyces*	0.5
						*Streptacidiphilus*	0.3
						*Kitasatospora*	0.2
				*Micromonosporaceae*	1.1	*Micromonospora*	0.3
						*Actinoallomurus*	0.2
						*Luedemannella*	0.3
				*Microbacteriaceae*	0.3	*Agromyces*	0.0
				*Thermomonosporaceae*	0.3	*Actinomadura*	0.1
				*Frankiaceae*	0.3	*Frankia*	0.3
				*Pseudonocardiaceae*	0.3	*Pseudonocardia*	0.2
				*Nocardiaceae*	0.1	*Marmoricola*	0.0
		*Solirubrobacterales*	2.5	*Solirubrobacteraceae*		*Solirubrobacter*	0.0
				*Patulibacteraceae*	0.1	*Patulibacter*	0.1
*Bacilli*	1.5			*Bacillaceae*	0.7	*Bacillus*	0.7
				*Paenibacillaceae*	0.6	*Paenibacillus*	0.5
*Gemmatimonadetes*	0.6			*Gemmatimonadaceae*	0.6	*Gemmatimonas*	0.3
*Chloroflexi*	2.4			*Ktedonobacteraceae*		*Ktedonobacter*	2.2
*Clostridia*	0.2	*Thermoanaerobacteriales*	0.1	*Thermodesulfobiaceae*	0.1	*Coprothermobacter*	0.1
*Nitrospirae*	0.2	*Nitrospirales*	0.2	*Nitrospiraceae*	0.2	*Nitrospira*	0.2
*Unknown*	0.4		15.6		37.6		57.5
*Bacteroidetes*	0.9	*Sphingobacteriales*	0.8			*Chitinophaga*	0.0
*Planctomycetes*	0.2	*Planctomycetales*	0.1			*Planctomyces*	0.0

The least abundant sequences of 454-pyrosequencing were represented by *γ*-*Proteobacteria* (4.1%), *δ*-*Proteobacteria* (3.5%), *β*-*Proteobacteria* (2.9%), *Chloroflexi* (2.4%), and *Bacilli* (1.5%). In the clone library, the least abundant sequences were represented by *β*-*Proteobacteria* (2.8%), *Chloroflexi* (2.1%), *Cyanobacteria* (2.8%), *Planctomycetes* (1.4%), *Bacilli* (0.7%), and *δ*-*Proteobacteria* (0.7%; Fig.3).

At a cutoff of 3% dissimilarity (i.e., 97% sequence identity) among the pyrosequencing reads, 12,621 OTUs were obtained from the 85,324 unique sequences (Table2). To estimate species richness, the ACE (abundance-based coverage), Boot, and Chao1 estimators were used. At the OTU cutoff of 3%, 12,621 OTUs were obtained from the 116,475 reads. The respective total numbers of species were estimated to be 23,489, 14,920, and 19,871 (Table2). At 3% dissimilarity, 73 OTUs were obtained from the 142 bacterial clone sequences. ACE, Boot, and Chao1 estimators for this dataset were 199, 92, and 189, respectively (Table2).

**Table 2:** Estimates of taxon richness and diversity indices for 16S rRNA gene-454-pyrosequencing and the clone library based on various evolutionary distance criteria for demarcating operational taxonomic units

Evolutionary distance	Richness				Diversity index	
	No. of OTUs	ACE	Boot	Chao1	Shannon	Simpson
Pyro seqs						
0.03	12,621	23,489	14,920	19,871	7.75	526
0.05	7500	10,718	8687	10,833	7.03	307
0.1	3126	4060	3535	4096	5.77	79
0.2	807	1016	899	1024	4.02	14
Clones						
0.03	73	199	92	189	3.95	48
0.05	60	165	75	130	3.52	21
0.1	40	80	49	69	2.72	7
0.2	18	29	21	28	1.64	3

OTU, operational taxonomic unit; ACE, abundance-based coverage estimator.

To determine richness based on pyrosequencing datasets and bacterial clone sequences, we identified 12,621, 7500, 3126, and 807 OTUs and 73, 60, 40, and 18 OTUs based on 3% (species level), 5% (genus level), 10% (family level), and 20% (phylum level) dissimilarity, respectively (Table2). Bacterial community composition based on 454-pyrosequences (3% dissimilarity) revealed that 12,621 OTUs were represented in the soil (Fig.2). The Shannon–Wiener (*H*) and reciprocal Simpson's (*1/D*) indices based on the clone library were 3.95 and 3.52 (*H*) and 48.1 and 20.8 (*1/D*) at 3% and 5% dissimilarity, while the *H* and *1/D* indices of pyrosequences were 7.75, 7.03 (*H*) and 526, 306 (*1/D*) at 3% and 5% dissimilarity, respectively. Chao1, based on the clone library and pyrosequences of the Gotjawal soil, was also higher than the samples (189, 130 and 19,871, 10,833) at the same dissimilarity. The diversity indices and richness of the Gotjawal soil bacteria were higher than Hawaiian volcanic deposits (Gomez-Alvarez et al. 2007) (Table2).

**Figure 4:**
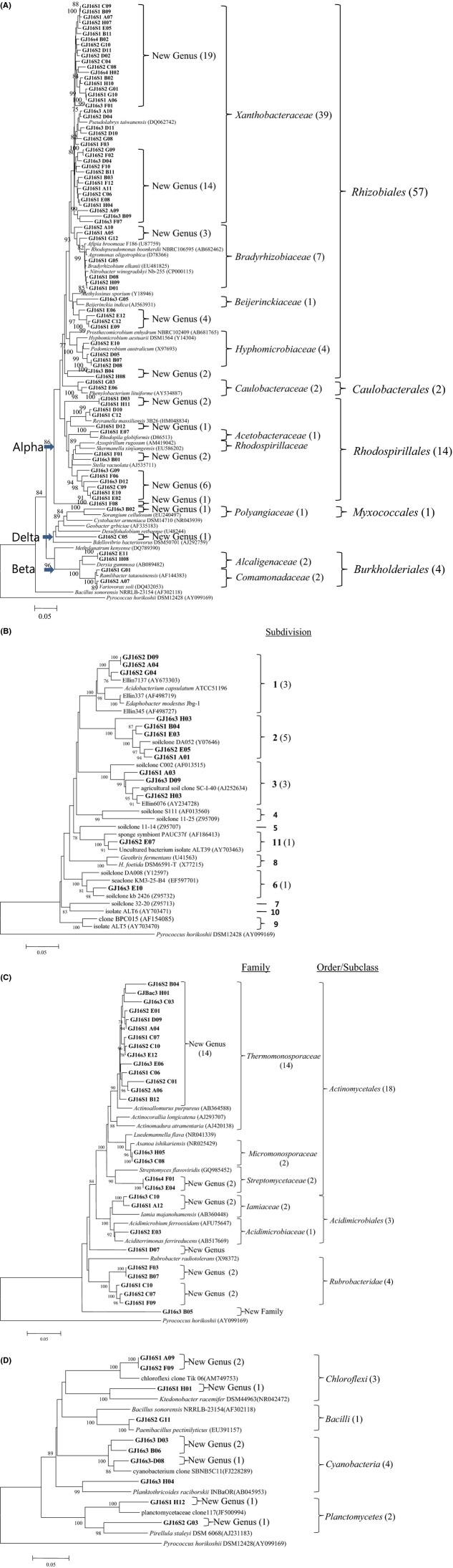
Phylogenetic tree of 142 bacterial clone sequences. (A) *Proteobacteria* from Gotjawal soils (80 clone sequences), with reference sequences. Twelve previously unknown clades are indicated as novel genera within the phylum. (B) *Acidobacteria* (13 clone sequences), with reference sequences. (C) *Actinobacteria* (28 clone sequences). (D) *Chloroflexi*, *Bacilli*, *Cyanobacteria*, and *Planctomycetes* (10 clone sequences). Bold letters refer to clone sequences. Each clade that appears to be a previously discovered taxon, based on the 16S rRNA divergence from the nearest previously known relative, is indicated.

Moreover, based on the comparison of the obtained 16S rRNA gene sequences to their closest known relatives, we discovered several new taxa at the species and genus levels. Figure4A shows the discovery of two novel genera within *δ*-*Proteobacteria* and 10 novel genera within *α*-*Proteobacteria*. Unexpectedly, in 454-pyrosequencing, *β*- (2.9% of OTUs), *γ*- (4.1% of OTUs), and *δ*-*Proteobacteria* (3.5% of OTUs) accounted for only low percentages of the total OTUs. Other orders were affiliated with *Caulobacterales* (0.6% of OTUs), *Rhodocyclales* (0.2%), and *Sphingomonadales* (0.1%; Table1).

### The order *Rhizobiales* of *α-Proteobacteria*

Among the *α*-*Proteobacteria* (45.7% of OTUs) represented in the 454-sequence dataset, the order *Rhizobiales* (30.2% of OTUs) was represented by the families *Hyphomicrobiaceae*, *Xanthobacteraceae*, *Rhodobiaceae*, *Bradyrhizobiaceae*, and *Rhizobiaceae*, with *Xanthobacteriaceae* as the core group (16.3%; Fig.4A). Overall, however, the phylotypes identified at the genus level were unknown. Within the family *Bradyrhizobiaceae* (4.8% of OTUs), the genus *Bradyrhizobium* accounted for 4.2% of the total OTUs, suggesting that this genus, and nitrogen fixers in general, may be major contributors to the Gotjawal nitrogen cycle. However, further careful studies are required to support this hypothesis.

The results of clone library analysis showed that *α*-*Proteobacteria* represented a high percentage of the total bacteria in the Gotjawal soil, which may be a key factor in the maintenance of the forest ecosystem (Table1). Different results were obtained with a forest soil, where *α*-*Proteobacteria* made up only approximately 11.4% of the total bacteria (Nacke et al. 2011). Recently, our research group reported a new genus, *Variibacter gotjawalensis* gen. nov., sp. nov., isolated from the soil of Aewol Gotjawal forest, one of four Gotjawal sites on Jeju Island (Kim et al. 2014b); this isolate belongs to *α*-*Proteobacteria* and shows high similarity with *Bradyrhizobium oligotrophicum* LMG 10732 (93.6%).

### Acidobacteria

Among the *Acidobacteria* identified by 454-pyrosequencing, *Acidobacteriales* (4.0% of OTUs), *Acidobacterium* (0.4% of OTUs), *Edaphobacter* (0.6% of OTUs), and *Candidatus Koribacter* (0.3% of OTUs) were affiliated with subdivision 1. *Bryobacter* (0.8% of OTUs) and *Candidatus Solibacter* (2.6% of OTUs) were affiliated with subdivision 3.

In a clone library analysis, the phylum *Acidobacteria* has been shown to occur in diverse environments as a dominant (up to 73%) bacterial group (Chan et al. 2006; Janssen 2006). However, in forest soil, *Acidobacteria* represent only 20.4% of total bacteria (35). The branched patterns of subdivisions 1, 2, and 3 were similar. From the 10 clones of the phylum *Acidobacteria*, five clades represented previously discovered subdivisions that were most closely related to subdivisions 1, 2, 3, 6, and 11 (Fig.4B).

### Actinobacteria

Among the *Actinobacteria* identified by 454-pyrosequencing, *Actinomycetales* (17.5% of OTUs) and *Solirubrobacterales* (2.5%) were the most abundant families, while *Acidothermus* was the largest genus at 11.5% of OTUs. *Mycobacterium* (2.0%) and *Micromonospora* (0.3%) were also readily detected.

Among the cloned sequences, *Actinobacteria* were dominant (21%). A phylogenetic analysis of these sequences revealed that the Gotjawal soil contained a variety of previously undiscovered genera and species of *Actinobacteria* (Fig.4C). We discovered six new clades at the genus level and one new clade at the family level.

Other phyla observed in the pyrosequencing dataset included *Ktedonobacter* (a genus in *Chloroflexi*, 2.2%) and *Gemmatimonas* (a genus in *Planctomyces*, 0.3%; Table1). *Cyanobacteria* (four clones) and *Chloroflexi* (two clones) were observed at low abundances in the clone library (Fig.4D).

### Functional bacterial genera

As Roesch et al. (2007) and Uroz et al. (2010) have suggested, and as is shown in Table3, the nitrogen-fixing bacteria primarily detected by 16S rRNA gene pyrosequencing belonged to *Bradyrhizobium* (4.2%) and *Rhizobium* (0.6%). A large number of sequences from the cellulolytic bacterial genus *Acidothermus* (Barabote et al. 2009) were also detected in the forest soil (Table1). In addition, few anaerobic AOB (0.002%) and methane-oxidizing bacteria (0.002–0.04%, *Methylocella* genus) were detected. However, 16S rRNA gene taxonomies were only loosely correlated with function.

**Table 3:** Number and percent of sequences classified to known functional bacterial genera based on the data derived from 454-pyrosequences

	Count	Percent
Nitrifying bacteria		
*Nitrospira*	144	0.16
*Nitrosospira*	3	0.003
Nitrogen-fixing bacteria		
*Rhizobium*	525	0.61
*Bradyrhizobium*	3556	4.16
*Mesorhizobium*	23	0.02
*Frankia*	234	0.27
Sulfur- and sulfate reducing bacteria		
*Geobacter*	5	0.005
Methane-oxidizing bacteria		
*Methylocystis*	6	0.007
*Methylocapsa*	2	0.002
*Methylocella*	41	0.04
*Methylobacterium*	4	0.004
Cellulolytic bacteria		
*Acidothermus*	9848	11.54

This study has some limitations. First, the dataset was small, and no replications were performed. This makes both the analysis and comparison with other studies difficult. Additionally, many of the identified sequences were not affiliated with known taxa. While this is a common occurrence, further studies are necessary to determine the importance of these results with regard to the ecology and characteristics of the Gotjawal forest. Analysis of the extent to which sequences from Gotjawal are completely novel, or whether these sequences have been observed in other soils or other systems, would also be of value. Further data are necessary to determine the novelty of these results. In addition, the presence of nitrogen-fixing bacteria is only important if we can document members of Fabaceae among the plants. Despite these limitations, this study is the first to analyze the bacterial communities within Gotjawal forest soils, and further studies are needed.

In conclusion, the high sequence identity of many of the bacterial clones to only environmental reference clones suggested that the majority of 16S rRNA gene pyrosequencing datasets and gene clones in Gotjawal soils were not affiliated with known genera or species. We discovered 18 novel genera and one novel family, as well as various novel species candidates, within the bacterial domain. The high rate of retrieval of new genus candidates (frequency of >50%) suggested that the communities may be highly specialized for growth in lava forest soils. Furthermore, the soil of the Gotjawal forest exhibited a unique bacterial composition containing unclassified *Actinobacteria* and *α*-*Proteobacteria*. Therefore, further work is necessary to fully elucidate the composition of the bacterial community and the functions of these soils.

## References

[b1] Amann RI, Ludwig W, Schleifer KH (1995). Phylogenetic identification and in situ detection of individual microbial cells without cultivation. Microbiol. Rev.

[b2] Barabote RD, Xie G, Leu DH, Normand P, Necsulea A, Daubin V (2009). Complete genome of the cellulolytic thermophile *Acidothermus cellulolyticus* 11B provides insights into its ecophysiological and evolutionary adaptations. Genome Res.

[b3] Chan OC, Yang X, Fu Y, Feng Z, Sha L, Casper P (2006). 16S rRNA gene analyses of bacterial community structures in the soils of evergreen broad-leaved forests in south-west china. FEMS Microbiol. Ecol.

[b4] Cole JR, Wang Q, Cardenas E, Fish J, Chai B, Farris RJ (2009). The Ribosomal Database Project: improved alignments and new tools for rRNA analysis. Nucleic Acids Res.

[b5] Crews TE, Kurina LM, Vitousek PM (2001). Organic matter and nitrogen accumulation and nitrogen fixation during early ecosystem development in Hawaii. Biogeochemistry.

[b6] Cutler NA, Chaput DL, van der Gast CJ (2014). Long-term changes in soil microbial communities during primary succession. Soil Biol. Biochem.

[b7] DeSantis TZ, Hugenholtz P, Keller K, Brodie EL, Larsen N, Piceno YM (2006a). NAST: a multiple sequence alignment server for comparative analysis of 16S rRNA genes. Nucleic Acids Res.

[b8] DeSantis TZ, Hugenholtz P, Larsen N, Rojas M, Brodie EL, Keller K (2006b). Greengenes, a chimera-checked 16S rRNA gene database and workbench compatible with ARB. Appl. Environ. Microbiol.

[b9] Dunfield KE, King GM (2004). Molecular analysis of carbon monoxide-oxidizing bacteria associated with recent Hawaiian volcanic deposits. Appl. Environ. Microbiol.

[b10] Finkel OM, Burch AY, Elad T, Huse SM, Lindow SE, Post AF (2012). Distance-decay relationships partially determine diversity patterns of phyllosphere bacteria on Tamrix trees across the Sonoran Desert. Appl. Environ. Microbiol.

[b11] Gomez-Alvarez V, King GM, Nusslein K (2007). Comparative bacterial diversity in recent Hawaiian volcanic deposits of different ages. FEMS Microbiol. Ecol.

[b12] Hernandez M, Dumont MG, Calabi M, Basualtoand D, Conrad R (2014). Ammonia oxidizers are pioneer microorganisms in the colonization of new acidic volcanic soils from south of Chile. Environ. Microbiol. Rep.

[b13] Huber T, Faulkner G, Hugenholtz P (2004). Bellerophon: a program to detect chimeric sequences in multiple sequence alignments. Bioinformatics.

[b14] Janssen PH (2006). Identifying the dominant soil bacterial taxa in libraries of 16S rRNA and 16S rRNA genes. Appl. Environ. Microbiol.

[b15] Jung SH (2009). Insects of Seonheul Gotjawal (covered by a rubble flow) in Jeju Island. J. Korean Nat.

[b16] Jung SH, Kim D S, Kim KJ (2010). Diversity of insect fauna. Study of geology, flora and fauna in Gotjawal Terrain II – Jocheon-Hamduck.

[b17] Kelly LC, Cockell CS, Piceno YM, Andersen GL, Thorsteinsson T, Marteinsson V (2010). Bacterial diversity of weathered terrestrial Icelandic volcanic glasses. Microb. Ecol.

[b19] Kim DS, Lee JH, Kim D S, Kim KJ, Yang SH (2010). Plant community dynamics. Study of geology, flora and fauna in Gotjawal Terrain II – Jocheon-Hamduck.

[b20] Kim JS, Makama M, Petito J, Park NH, Cohan FM, Dungan RS (2012). Diversity of bacteria and archaea in hypersaline sediment from Death Valley National Park, California. MicrobiologyOpen.

[b21] Kim JS, Jung MY, Lee KC, Kim DS, Ko SH, Lee JS (2014a). The Archaea community associated with lava-formed Gotjawal forest soil in Jeju, Korea. J. Agr. Chem. Environ.

[b22] Kim KK, Lee KC, Eom MK, Kim JS, Kim DS, Ko SH (2014b). *Variibacter gotjawalensis* gen. nov., sp. nov., isolated from soil of a lava forest. Antonie Van Leeuwenhoek.

[b23] Kimura M (1980). A simple method for estimating evolutionary rates of base substitutions through comparative studies of nucleotide sequences. J. Mol. Evol.

[b24] King GM (2003). Contribution of atmospheric CO and hydrogen uptake to microbial dynamics on recent Hawaiian volcanic deposits. Appl. Environ. Microbiol.

[b25] King CE, King GM (2012). Temperature responses of carbon monoxide and hydrogen uptake by vegetated and unvegetated volcanic cinders. ISME J.

[b26] King GM, Weber CF (2008). Interactions between bacterial carbon monoxide and hydrogen consumption and plant development on recent volcanic deposits. ISME J.

[b27] Lane DJ, Stackebrandt E, Goodfellow M (1991). 16S/23S rRNA sequencing. Nucleic acid techniques in bacterial systematics.

[b28] Li W, Fu L, Niu B, Wu S, Wooley J (2012). Ultrafast clustering algorithms for metagenomic sequence analysis. Brief Bioinform.

[b29] Nacke H, Thurmer A, Wollherr A, Will C, Hodac L, Herold N (2011). Pyrosequencing-based assessment of bacterial community structure along different management types in German forest and grassland soils. PLoS One.

[b30] Nanba K, Kung GM, Dunfield K (2004). Analysis of facultative lithotroph distribution and diversity on volcanic deposits by use of the large subunit of ribulose 1,5-bisphosphate carboxylase/oxygenase. Appl. Environ. Microbiol.

[b31] Nusslein K, Tiedje JM (1998). Characterization of the dominant and rare members of a young Hawaiian soil bacterial community with small-subunit ribosomal DNA amplified from DNA fractionated on the basis of its guanine and cytosine composition. Appl. Environ. Microbiol.

[b32] Ohta H, Yagi M, Suzuki J, Fujitake N, Watanabe M (2003). Characterization of *Sphingomonas* species found as predominant members in the culturable bacterial community of a green pigment-containing sclerotium grain from Mt. Myoko (Japan) volcanic ash soil. Microbes Environ.

[b33] Oksanen J, Guillaume Blanchet F, Kindt R, Legendre P, Minchin PR, O'Hara RB (2011). http://CRAN.R-project.org/package=vegan.

[b34] Park JB, Kim D S, Kim KJ (2010). Characterization of lava-forming petrology and petrochemistry. Study of geology, flora and fauna in Gotjawal Terrain II – Jocheon-Hamduck.

[b35] Pruesse E, Quast C, Knittel K, Fuchs BM, Ludwig W, Peplies J (2007). SILVA: a comprehensive online resource for quality checked and aligned ribosomal RNA sequence data compatible with ARB. Nucleic Acids Res.

[b36] Roesch LF, Fulthorpe RR, Riva A, Casella G, Hadwin AK, Kent AD (2007). Pyrosequencing enumerates and contrasts soil microbial diversity. ISME J.

[b37] Schloss PD, Handelsman J (2005). Introducing DOTUR, a computer program for defining operational taxonomic units and estimating species richness. Appl. Environ. Microbiol.

[b38] Schloss PD, Westcott SL, Ryabin T, Hall JR, Hartmann M, Hollister EB (2009). Introducing MOTHUR: open source, platform independent, community-supported software for describing and comparing microbial communities. Appl. Environ. Microbiol.

[b39] Tamura K, Peterson D, Peterson N, Stecher G, Nei M, Kumar S (2011). MEGA5: molecular evolutionary genetics analysis using maximum likelihood, evolutionary distance, and maximum parsimony methods. Mol. Biol. Evol.

[b40] Torsvik V, Goksoyr J, Daae FI (1990). High diversity in DNA of soil bacteria. Appl. Environ. Microbiol.

[b41] Uroz S, Buee M, Murat C, Frey-Klett P, Martin F (2010). Pyrosequencing reveals a contrasted bacterial diversity between oak rhizosphere and surrounding soil. Environ. Microbiol. Rep.

[b42] Yang KS, Kim SB, Kim SY, Lee GE, Kim WT (2006). Community analysis of the moths in the Gotjawal terrains of Jeju Island, Korea. J. Ecol. Field Biol.

